# Lithium Chloride can Induce Differentiation of Human Immortalized RenVm Cells into Dopaminergic Neurons

**Published:** 2017

**Authors:** Mitra Soleimani, Nazem Ghasemi

**Affiliations:** Department of Anatomical Sciences and Molecular Biology, Faculty of Medicine, Isfahan University of Medical Sciences, Isfahan, Iran

**Keywords:** Beta catenin, Cell differentiation, Lithium, Wnt proteins

## Abstract

**Background:**

Stem cell-based therapy is a novel strategy for the treatment of neurodegenerative diseases. The transplantation of fully differentiated cells instead of stem cells in order to decrease serious adverse complications of stem cell therapy is a new idea. In this study, the effect of lithium chloride on dopaminergic differentiation of human immortalized RenVm cells was investigated in order to access a population of fully differentiated cells for transplantation in Parkinson disease.

**Methods:**

The immortalized RenVm cells were induced to dopaminergic differentiation using a neurobasal medium supplemented with N2 and different concentrations (1, 3, 6 *mM*) of Lithium Chloride (LiCl) for 4, 8 and 12 days. The efficiency of dopaminergic differentiation was evaluated using immunocytochemistry and western blot techniques for tyrosine hydroxylase and β-catenin marker expression.

**Results:**

Our results indicated that LiCl can promote dopaminergic differentiation of RenVm cells in a dose-dependent manner.

**Conclusion:**

It can be concluded that LiCl is able to facilitate dopaminergic differentiation of cultured cells by affecting Wnt-frizzled signaling pathway.

## Introduction

The conventional therapies for neurodegenerative diseases are based on either immunomodulation/anti inflammation or neurotransmitter replacement ^1–4^. Although these pharmacological therapies can partially reverse neuronal disturbances, these methods result in some side effects. Recently, cell-based therapy is proposed as a novel paradigm for treatment of neurodegenerative diseases such as multiple sclerosis ^5^, Parkinson ^6^, Huntington’s ^7^ and Alzheimer’s disease ^8^. Although the principal mechanism responsible for these therapeutic effects of stem cell transplantation is not clear, but it seems to be due to trophic effects and differentiation potential of stem cells into functional neurons ^5^, ^9^.

Unlike the studies which offered the beneficial potential of stem cell therapy, the serious adverse events of this method such as tumorigenic potential cannot be denied. The differentiation degree of the transplanted cells is one of the most important factors which is involved in tumorigenesis. Therefore, molecular pathways involved in signaling cell differentiation have been studied by many researchers in order to access a population of fully differentiated cells.

Wnt proteins are a group of cysteine-rich glycosylated proteins that are able to stimulate the growth of specific tissues and play an important role in the regulation of various cellular processes such as cell proliferation and differentiation ^10–12^.

Wnt1 is a class of these proteins which consists of several members including Wnt1, Wnt2, Wnt3, Wnt8 proteins ^13^. The biological activity of these proteins is mediated via specific receptors, including the frizzled transmembrane receptor and lipoprotein related protein 5 and 6 (LRP-5/6) ^14^. According to current published data, Wnt1 proteins when binding to their receptors are able to activate another intracellular pathway termed canonical Wnt/ß-catenin pathway which leads to inhibition of the downstream glycogen synthase kinase-3ß (GSK-3ß). Thus, ß-catenin phosphorylation, ubiquitination and subsequent degradation thorough proteasomes will be suppressed. As a result, ß-catenin can translocate into the cell nucleus and bind to DNA which can trigger gene transcription. Overall, several particular biological functions such as specific precursor cells regulation, cellular differentiation and nervous tissue development may occur through canonical Wnt-frizzled signaling pathway ^15–18^. Therefore, the induction of cell differentiation is possible using specific factors with the potential to trigger Wnt-Frizzled signaling pathway.

Lithium Chloride (LiCl) is an agent which is widely used to treat manic depressive illness ^19,20^. This chemical compound can affect the central nervous system in a variety of ways including inhibition of glycogen synthase kinase 3 β (GSK3β) and mimicking the effects of Wnt signaling on gene expression, cell proliferation and differentiation ^21–23^. In addition, lithium possesses additional beneficial effects including neuroprotective effects through increasing the brain-derived neurotrophic factor ^24,25^ and vascular endothelial growth factor expression ^26,27^, anti-apoptotic effects by inducing up-regulation of B-cell lymphoma protein-2 (bcl-2) as well as suppressing the calcium-dependent activation of pro-apoptotic signaling pathways ^28,29^.

An immortalized cell line refers to a population of undifferentiated cells that are characterized by high self-renewal ability and multi-potency. Thus, these cells can be grown in vitro for long periods and can create more cells. In addition, these cells are capable to differentiate into other cells lineage such as neurons, astrocytes and oligodendrocytes. Therefore, these cells are very important tools for research in cell biology and cell based therapy. RenVM is one of the immortalized human neural stem cell lines which is isolated from 10 week fetal neural ventral mesencephalon and was established with the V-myc oncogene by retroviral transduction.

With respect to the broad beneficial effects of lithium in signaling cell pathways, in the current study, the effects of several doses of lithium chloride were evaluated on dopaminergic differentiation of human immortalized RenVm cells.

## Materials and Methods

### RenVm cells culture

All chemicals, unless specified otherwise, were purchased from Sigma-Aldrich, St. Louis, MO, USA. In addition, the human immortalized RenVm cell lines were purchased from the Regeneron company. The immortalized RenVm cells were seeded at the concentration of 25,000 cell/CM2 in 25 *cm*^2^ flasks and propagated in the pre-differentiation medium consisting of Dulbecco’s Modified Eagles Medium (DMEM) supplemented with B27 (gibco), gentamicin 50 *ng/ml*, bFGF 10 *ng/ml*, EGF 20 *ng/ml*, and heparin 10 *U/ml* in a 37*°C* humidified incubator with a 5% CO_2_ environment. At approximately 80% confluency, these cells were passaged and then seeded at approximately 10000 cells /CM2 in laminin coated 96 wells.

### Dopaminergic differentiation of RenVm cells

Differentiation stage was initiated by discharge of growth factors once the cells reached a confluency of 90%. Immortalized RenVm cells were dissociated using trypsin (500 *μg/ml*) solution and seeded at a density of 10000 *cells/cm*^2^ on laminin coated 96 wells. Cells were maintained in differentiation medium comprised of neurobasal medium (gibco) supplemented with N2 (gibco), gentamicin, and different concentrations (1, 3, 6 *mM*) of LiCl for 4, 8 and 12 days. For control group, cells were maintained in differentiation medium without lithium. In addition, for Control-DMSO (C-DMSO) cells were maintained in differentiation medium supplemented with 0.1% DMSO. The media changed every other day with the ratio of 80%. The cells were fixed on days 4, 8 and 12 for further experiments.

### Immunocytochemistry

Four, eight and twelve days after neural induction, the cells were fixed with 4% paraformaldehyde for half an hour. The cells were permeabilized with 10% *v/v* normal donkey serum in PBS-Triton 0.3% *v/v* for 1 *hr* at room temperature. Fixed cells were washed 3–4 times in PBS and were incubated with primary antibodies (mouse anti TH, 1:500 dilution; anti TuJ1, 1:1000 dilution) overnight at 37*°C*. After washing with PBS, the cells were exposed to 1 *hr* with a 1:100 dilution of Alexa 488-conjugated goat anti mouse IgG secondary antibody. Finally, cells were observed using fluorescence microscope (Nikon Inc., Melville, NY).

For quantitative analysis, total number of positive cells was counted on each acquired image by ImageJ, and the ratio to the number of nuclei was analyzed for each antigen and the number of immunopositive cells was counted in a minimum total of 200 cells per slide. In addition, all immunocytochemistry studies were repeated at least twice.

### Western blot

Differentiated cells were subjected to Western blot analysis. Briefly, the samples were lysed using lysis buffer, centrifuged at 14,000 *g* for 30 *min* at 4*°C* and the supernatant was used for immunodetection with anti TH (1:500 dilution), anti TuJ1(1 *μg/ml*) and anti β-catenin (0.25 *μg/ml*). Finally appropriate secondary antibodies conjugated with alkaline phosphatase were used and the relative expression levels of TH, TuJ1and β-catenin proteins were assessed. In addition, all western blot analysis was repeated at least twice.

## Results

### RenVm cells characterization before and after differentiation

**Figure 1.**
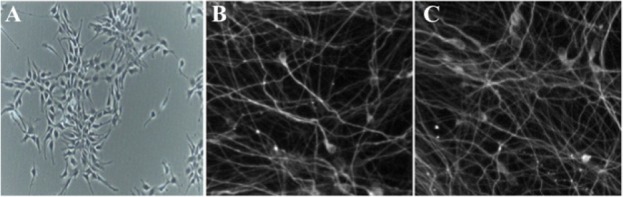
Phase contrast and fluorescence images of cultured and differentiated RenVm cells. RenVm cells at the beginning of the neural differentiation (A). The differentiated RenVm cells four (B) and eight (C) days after the beginning of differentiation. Scale bars represent 200 *μm* in A and 100 *μm* in B and C.

The morphology of the RenVm cells depended on mitogenic factors (EGF and FGF-2) used for their expansion. The cells expanded as islands (or clusters) of cells, and appeared to have bipolar cell morphology (Figure 1A). Under growth conditions, they displayed an undifferentiated neural morphology. In addition, they showed rosette-like formations when reaching confluency. After differentiation, ReNcell VM cells formed networks in which initial extensions elongated and became more complex leading to a dense cellular network. Over time, ReNcell VM cells started to form more complex cell connections (Figures 1B and 1C).

### Immunocytochemistry study of dopaminergic differentiation

**Figure 2.**
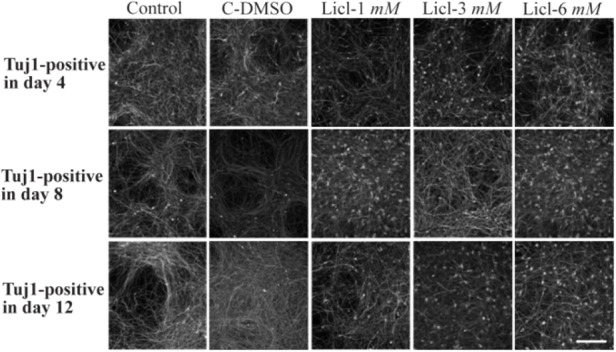
Immunocytochemistry images of differentiated cells which expressed Tuj1 marker in different concentrations of lithium (1 *mM*, 3 *mM*, 6 *mM*), control and in DMSO control (C-DMSO) groups. Scale bar represents 100 *μm.*

**Figure 3.**
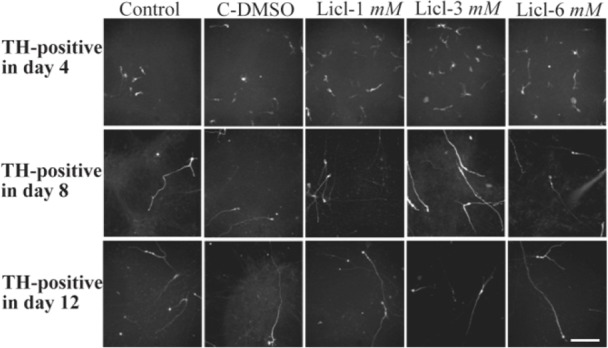
Immunocytochemistry images of differentiated cells which expressed tyrosine hydroxylase marker in different lithium concentrations (1, 3, 6 *mM*), control and in DMSO control (C-DMSO) groups. Scale bar represents 100 *μm.*

**Figure 4.**
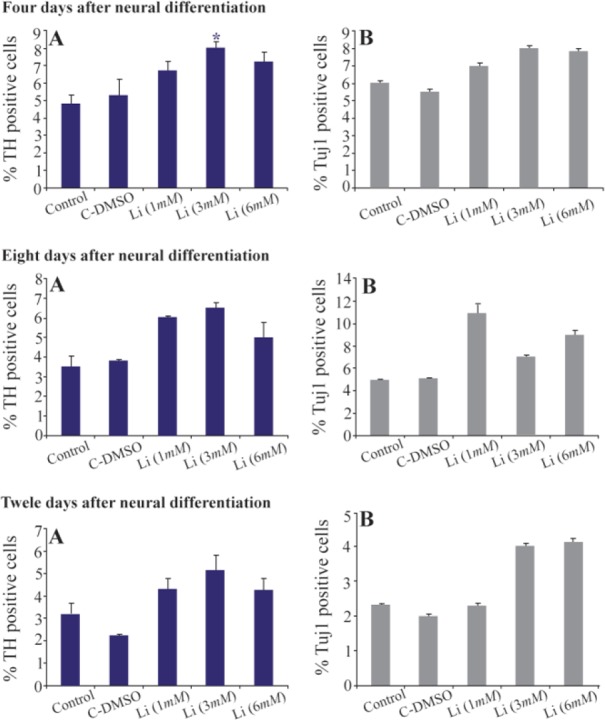
The mean percentage of differentiated cells which expressed TH and Tuj1 markers. In the 3 *ng/ml* concentration of lithium on the fourth day, the mean percentage of TH positive cells significantly increased compared to other groups (p≤0.05)

Immunocytochemistry staining with cell type-specific markers was used to recognize the phenotype of differentiated cells. Fluorescence microscopic analysis four, eight and twelve days post neural induction showed that the mean percentage of cells which expressed TH and Tuj1 markers was different in all of the studied groups (Figures 2 and 3). In addition, in one of the lithium concentrations (3 *ng/ml*) on the fourth day, a high percentage of cells expressed TH marker which was significant when compared with other groups (Figure 4).

### Western blot analysis

**Figure 5.**
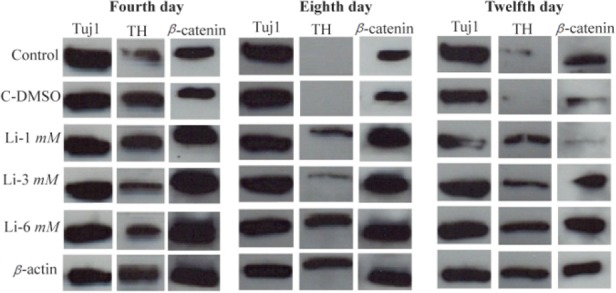
Western analysis of differentiated cells in different concentrations of LiCL for TH, Tuj1 and β-catenin markers in 4, 8 and 12 days. β-actin was used as a control marker.

Four, eight and twelve days post neural induction, protein was extracted from differentiated cells for western blot assays. To this end, β-actin was used as a control marker. Our finding showed that the expression of TuJ1 marker was almost identical in all groups, but in line with immunocytochemistry results, the expression of TH in 3 *ng/ml* of lithium on the fourth day was also higher when compared to other groups (Figure 5).

## Discussion

There is a lot of concern about the safety of cell-based therapy in treatment of neurodegenerative diseases ^30^. In order to reduce the side effects of this procedure, the transplantation of fully differentiated cells instead of stem cells has been suggested. To this end, the present study was done in order to promote human immortalized RenVm cells differentiation into dopaminergic neuron using LiCl. LiCl is one of the important factors which could alter gene expression through affecting cellular signaling and thus determine cell fate ^21–23^. The results of present study revealed that LiCl can promote differentiation of RenVm cells and induce the expression of TH in cultured cells. As shown in figure 4, the induction of the TH expression in RenVm cells is dose-dependent and time-dependent. In particular, 3.0 *mM* LiCl administration after four days showed the highest effect on TH marker expression than other LiCl concentrations. Additionally, western blot results demonstrated that TH expression level increased in all groups treated with LiCl especially in four days. β-catenin expression level also increased in the groups treated with LiCl compared to the control and DMSO groups. Therefore, a hypothesis can be considered in which LiCl may participate in promotion of dopaminergic differentiation by inhibition of the GSK-3ß activity. As a result, the inhibition of β-catenin phosphorylation occurs and the cytoplasmic level of this factor increases. In conclusion, β-catenin can enter the nucleus and by binding to DNA, is able to increase the transcription of specific genes involved in dopaminergic differentiation.

## Conclusion

Overall, it can be concluded that LiCl is able to affect a variety of cell signaling pathways such as Wnt-Frizzled signaling pathway. This agent within the therapeutic range is able to inhibit both GSK-3ß activity and β-catenin phosphorylation and facilitates differentiation of RenVm cells to dopaminergic neuronal cells.
